# Machine Methods of Defense

**Published:** 1910-01

**Authors:** G. Frank Lydston

**Affiliations:** Chicago, Ill.


For Texas Medical Journal.

### Machine Methods of Defense.

BY G. FRANK LYDSTON, M. D., CHICAGO, ILL.

The tactics of the present political machine of the A. M. A. in defending itself against attack are very suggestive to one of the reflective turn of mind. Its policy seems to be one of gum shoes and darkness. Has the profession noted that the machine has done nothing but throw mud, avoid issues, and attempt to stifle truth? Is the machine so rock-ribbed that it considers itself impregnable? Does it fancy that the rank and file are all fools? Does it believe that the membership at large at "asleep at the switch"? What does the machine suppose has been the effect of the Mississippi Valley Association episode at St. Louis and the Ohio Valley Association episode at Evansville on the profession? If the machine does not realize that these occurrences have removed the blinders from the work horses who are pulling the A. M. A. band wagon it has even less sense than I had given it the credit of possessing—and that was little enough.

Did not the reception accorded me at Columbus, Ohio, and the enthusiasm with which my paper was received at the Ohio Valley Association point to the hand-writing on the wall? Apropos of the banquet tendered me at Columbus, why did not the local machine men turn out and in a manly way attack my paper? They received a cordial invitation to be present. Their absence surely was not due to a lack of interest in the subject, as was proven by the lying anonymous interviews attacking me which appeared in one of their prominent dailies.

That they considered me a formidable foe who must be dealt with by knavery was shown by the appearance of my picture in connection with a patent medicine ad. in the same paper the following day. I trust that the ample front page apology rendered me by the paper a few days later was consoling to the contemptible coyotes who anonymously sent me clippings of the ad.

The Columbus newspaper ad. episode was so despicable that I thought it must forever remain in a class by itself. It, however, remained for the editor of the *Texas State Journal of Medicine* to outdo the infamy of the Columbus machine crowd.

During the battle which I had with the machine men who are running the M. V. A. there was but one St. Louis newspaper that was unfriendly to me. This paper was unfriendly in a manner which plainly showed the machine source of its animus.

In a subsequent issue of this paper appeared a badly garbled interview with me on various sociologic problems. So garbled was this article that any man of even scant knowledge of sociology and of my own work in that field should have known at sight that much of the interview was not authentic.

Among other subjects I discussed the race problem in relation to miscegenation laws and the possible and probable effects of amalgamation of races. Not content with garbling the article, the paper supplied it with headlines which did not fit even the garbled text. These headlines stated that I advocated intermarriage of negroes and whites.

The profession of the South, and, indeed, of the entire country, is so familiar with my views upon the race problem, that I was astounded when I saw the entire garbled interview, glaring headlines and all, reproduced in the November number of the *Texas State Journal of Medicine*. The editorial contained comments which plainly showed the animus and ignorance of the man who wrote it.

On reading the editorial in question I immediately wrote the editor of the *Texas State Journal*, expressing my surprise that the editor of a scientific publication should quote as authentic a newspaper article on a scientific subject, and especially an article reflecting on a reputable member of the profession. I suggested that he of course would do the gentlemanly thing and publish my real views of the subjects touched upon in the editorial. To this letter he has made no reply. I also caused to be sent to him publications containing recent articles written by me upon the race problem. Among other publications containing my views which I requested the publisher to send him was Ex-Governor Vardaman's *Issue*, published at Jackson, Miss. As I have had letters commendatory of the article from other editors to whom I requested that it be sent, I infer that the editor of the *Texas State Journal of Medicine* received it. If he did not receive the *Issue*, he had abundant opportunity to read my views as published in many of his medical exchanges.

Not having received any expression of apology or desire to rectify his mistake and correct the misrepresentation of my views contained in his editorial, I can arrive at but one conclusion, viz., that the editor of the *Texas State Journal of Medicine* quoted the garbled newspaper article for the purpose of discrediting me and the cause of medico-political freedom which I represent, in the eyes of my Southern friends, whose name I am proud and happy to say is legion.

So firm is my faith in the friendships I have made in the South during my thirty years of labor in the medical vineyard, so sure am I that no differences of policies or politics can ever shake the foundations of those friendships, that I am confident that such malevolent shafts of falsehood as that aimed at me by the editor of the *Texas State Journal of Medicine* will fall harmless to the ground. Nay, more, they will rebound like a boomerang and wound the hand that threw them. Trust the chivalrous spirit of the true Southerner for that.

And, speaking of the true Southerner—surely the editor of the *Texas State Journal of Medicine* is not to the manner born. He must be alien to the great Lone Star State. If so, his Northern compatriots should hasten to disabuse the minds of their Southern friends of the notion that he represents Northern gentility. The true gentleman is the same on either side of the line, hence the editor of the *Texas State Journal of Medicine* would be *declasse* in any locality where gentility is to be expected. Surely he does not represent the spirit of the profession of the great State of Texas! If he does, then I am a poor judge of human nature, for I know men in the Texas profession—and many of them—on whose manliness and fair-mindedness I would wager a pretty penny. And not all of them agree with me in my medico-political views at that. On the contrary, some of them and I gaze at each other and wonder why the other fellow is so blind.

I am curious to know whether the profession of Texas will stand for an official representative whose only ammunition in a political campaign is mud, lies, slander and deliberate misrepresentation. Long ere the next meeting of the Texas State Medical Association the profession will have learned and acknowledged, not only that I am right, but that conditions in the A. M. A. are blacker than my feeble pen ever could portray. Then will the editor of the *Texas State Journal of Medicine* sink in the stew of machine corruption.

---

New Monograph on Hookworm Disease.—Announcement is made that a volume on hookworm disease, its etiology, pathology, prevention and treatment, will shortly appear from the pen of Drs. George Dock and Charles C. Bass, of Tulane University, New Orleans. The fact that there at least two million people in the United States afflicted with the disease, leaves no doubt that there is both a great field and a pressing need for a book on the subject. The C. V. Mosby Medical Book and Publishing Co., of St. Louis, will have charge of its publication.

